# Global Metabolomics Reveals That *Vibrio natriegens* Enhances the Growth and Paramylon Synthesis of *Euglena gracilis*

**DOI:** 10.3389/fbioe.2021.652021

**Published:** 2021-03-31

**Authors:** Ying Ouyang, Shuyu Chen, Liqing Zhao, Yiting Song, Anping Lei, Jiayi He, Jiangxin Wang

**Affiliations:** ^1^Shenzhen Key Laboratory of Marine Bioresources and Eco-Environmental Science, Shenzhen Engineering Laboratory for Marine Algal Biotechnology, Guangdong Provincial Key Laboratory for Plant Epigenetics, College of Life Sciences and Oceanography, Shenzhen University, Shenzhen, China; ^2^College of Chemistry and Environmental Engineering, Shenzhen University, Shenzhen, China; ^3^Department of Microbiology, University of Illinois Urbana-Champaign, Champaign, IL, United States; ^4^Key Laboratory of Optoelectronic Devices and Systems of Ministry of Education and Guangdong Provinces, College of Physics and Optoelectronic Engineering, Shenzhen University, Shenzhen, China

**Keywords:** *Euglena gracilis*, *Vibrio natriegens*, co-cultivation, metabolomics, paramylon

## Abstract

The microalga *Euglena gracilis* is utilized in the food, medicinal, and supplement industries. However, its mass production is currently limited by its low production efficiency and high risk of microbial contamination. In this study, physiological and biochemical parameters of *E. gracilis* co-cultivated with the bacteria *Vibrio natriegens* were investigated. A previous study reports the benefits of *E. gracilis* and *V. natriegens* co-cultivation; however, no bacterium growth and molecular mechanisms were further investigated. Our results show that this co-cultivation positively increased total chlorophyll, microalgal growth, dry weight, and storage sugar paramylon content of *E. gracilis* compared to the pure culture without *V. natriegens*. This analysis represents the first comprehensive metabolomic study of microalgae-bacterial co-cultivation, with 339 metabolites identified. This co-cultivation system was shown to have synergistic metabolic interactions between microalgal and bacterial cells, with a significant increase in methyl carbamate, ectoine, choline, methyl N-methylanthranilate, gentiatibetine, 4R-aminopentanoic acid, and glu-val compared to the cultivation of *E. gracilis* alone. Taken together, these results fill significant gaps in the current understanding of microalgae-bacteria co-cultivation systems and provide novel insights into potential improvements for mass production and industrial applications of *E. gracilis*.

## Introduction

*Euglena gracilis* is a single-celled flagellate alga with characteristics typical of both plants and animals, including the lack of a cell wall. Additionally, *E. gracilis* possesses two flagella for cell mobility (Zakryś et al., 2017). *E. gracilis* cells are rich in minerals, amino acids, unsaturated fatty acids, lutein, chlorophyll, zeaxanthin and 59 other essential nutrients for human health. Furthermore, it can be used to produce many valuable products, such as α-tocopherol, wax esters and paramylon (Kottuparambil et al., 2019). However, the low production efficiency in the large-scale cultivation of *E. gracilis* limits its further development. We therefore sought to determine whether the novel approach of utilizing co-cultivation would improve the efficacy of *E. gracilis* production.

There are several ecological mechanisms, such as quorum sensing, which occur among microbial communities to facilitate cell-cell communication (Zhou et al., 2016). The microalgae-bacteria co-cultivation system involves a mixture of microalgae and bacteria at specific ratios with the intent to increase the production of microalgae or specific substances by microalgae (Taniguchi and Tanaka, 2004). Such co-cultivation systems have been studied extensively. For instance, it has been found that the microbial interaction between the bacterium *Azospirillum brasilense* and the microalga *Chlorella vulgaris* has a significant effect on fatty acid and lipid accumulation in the microalga (Leyva et al., 2014). Additionally enhancement of microalgal growth, lipid and protein content of *Chlorella variabilis* was shown when the green microalga was co-cultivated with the siderophore-producing bacterium, *Idiomarina loihiensis* RS14, in an optimized ratio under iron-deficient conditions (Rajapitamahuni et al., 2019). When microalga *Chlorella* was co-cultured with bacteria *Bacillus firmus* and *Beijerinckia fluminensis* to treat wastewater in vinegar production, though algal biomass was slightly decreased, a higher rate of nutrients removal was achieved (Huo et al., 2020). Earlier work in *E. gracilis* reported that *Vibrio natriegens* can increase its biomass accumulation (Kim et al., 2019). Previous reports have also indicated that the biomass and paramylon production of *E. gracilis* is increased when it is co-cultivated with the bacterium *Pseudoalteromonas* under optimal conditions, the extracellular polymeric substances (EPS) of the bacterium contributed to the results (Jeon et al., 2019, 2020). When co-cultivated with the microalga growth-promoting bacterium *Emticicia*, *E. gracilis* was found to have higher biomass and produce more lipids (Toyama et al., 2019). Despite all of the beneficial effects found in these co-cultivation systems, the underlying mechanisms have not been sufficiently studied.

Metabolomics is the study of the physiology of organisms by profiling the changes in metabolites under different conditions. As downstream products biosynthesized after complex transcriptional, translational and regulatory processes, the types and amounts of metabolites vary significantly depending on different conditions (Nicholson et al., 1999). Thus, metabolomics has been applied to obtain a general understanding of the regulatory networks involved in microalgal metabolism. A metabolic profiling technique was developed for the model green microalga, *Chlamydomonas reinhardtii*, under stress conditions such as nitrogen-, phosphorus-, sulfur- and iron-depletion (Bölling and Fiehn, 2005). A method to assess the metabolism of freshwater microalga *C. vulgaris* and *Scenedesmus obliquus* after being exposed to the flame retardant triphenyl phosphate was also recently established (Wang et al., 2019). Furthermore, potential biomarkers of *C. reinhardtii* grown in photobioreactors in the context of nitrogen starvation were found using metabolomics (Courant et al., 2013).

In this study, we reproduced the previous finding that *V. natriegens* enhances both growth and paramylon production of *E. gracilis* (Kim et al., 2019). What’s more, we employed metabolomics in culture medium to gain a better understanding of the underlying causes of the improvements observed in the co-cultivation experiment, which revealed several interesting interactions between the two species since, and the results will be easily applied to large-scale exploitation for *E. gracilis* in the future. This is the first report utilizing culture medium metabolomics to understand this co-cultivation system, and therefore, fills a gap in the current understanding of microalgae-bacteria symbiosis on a metabolomic level.

## Materials and Methods

### Strains and Culture Conditions

*Euglena gracilis* CCAP 1224/5Z was purchased from the Culture Collection of Algae and Protozoa^1^. The microalgal cells were grown under a continuous light at a light intensity of approximately 100 μmol/m^2^/s in an illuminating incubator at 26°C in EM medium [1.8 g/L NH_4_Cl, 0.6 g/L KH_2_PO_4_, 0.6 g/L MgSO_4_, 60 mg/L Urea, 0.02g/L CaCl_2_, 0.48 mg/L Na_2_EDTA, 2 mg/L Fe_2_ (SO_4_)_3_, 60 μL HCl, 0.01 mg/L Vb_1_, 0.0005 mg/L Vb_12_, 20 mg/L CuSO_4_⋅5H_2_O, 0.4 g/L ZnSO_4_⋅7H_2_O, 1.3 g/L Co (NH_3_)⋅H_2_O, and 1.6 g/L MnCl_2_⋅4H_2_O] until reaching stationary phase. Subcultures of alga were done every 6 days at a ratio of 10% (Afiukwa and Ogbonna, 2007).

*Vibrio natriegens* 1H00025 was purchased from the Third Institute of Oceanography, MNR (Xiamen, China). The bacterial cells were grown in sterilized 2216E (CM0471) medium and incubated at 26°C with rotational shaking (120 r/min) in the dark. Subcultures of alga were done every 3 days at a ratio of 1% (Weinstock et al., 2016).

### Co-cultivation of *E. gracilis* and *V. natriegens*

*Euglena gracilis* cells were harvested during the stationary phase when the OD_750_ reached 3.0. The OD_750_ of each initial inoculum was adjusted to 3.0 after being washed three times in EG medium (Afiukwa and Ogbonna, 2007). *V. natriegens* cells were harvested during the exponential phase when OD_600_ reached 1.0. The OD_600_ of the initial inoculums were adjusted to 1.0 after being washed three times in EG medium. The inoculation volume ratio of *E. gracilis* to *V. natriegens* was 10:1 (200 mL: 20 mL). After mixing the two inoculums, additional medium was added to reach a final volume of 1.5 L. After dilution, co-cultivation of *E. gracilis* and *V. natriegens* was performed in an illuminating incubator at 26°C in EG medium under a continuous light at a light intensity of approximately 100 μmol/m^2^/s, no shaking or aeration was used during experiment.

### Growth (Cell Number, Chlorophyll Content, Dry Weight) and Paramylon Content of *E. gracilis*

The growth of *E. gracilis* was calculated by measuring its cell number, total chlorophyll content, and dry weight. Paramylon content was also calculated. Cell growth was measured by counting cell number with a microscope in a 0.1 mL counting chamber. The total chlorophyll of a 1 mL sample was extracted with 80% acetone and the chlorophyll content was determined using the Arnon method (Arnon, 1949). The dry weight of the 100 mL sample was measured using the oven-drying method (Edmunds, 1966). Paramylon extraction and measurement was carried out as reported previously (Takenaka et al., 1997). Cell number and total chlorophyll were measured daily over the course of 9 days. Dry weight and paramylon content were measured every two days. Three biological replicates were used for each experiment.

### The Cell Number of *V. natriegens*

The cell number of *V. natriegens* was measured manually with a microscope in a 0.1 mL counting chamber. Samples were first stained with crystal violet (Kannan et al., 2019) at a 9:1 ratio of sample to crystal violet for 15 minutes. Next, 100 μL of the stained sample was added into the counting chamber and completely fixed on the chamber surface after being dried in an oven.

### Preparation of Samples for LC-MS/MS Analysis

For LC-MS/MS analysis, samples taken after six days of incubation were first centrifuged at 5,000 g for 5 min at 4°C (Thermo Heraeus Fresco17, United States) to obtain supernatant. After centrifugation, 300 μL aqueous methanol (1 μg/mL of the inner label) was plunged into 100 μL of each supernatant sample, followed by vortexing for 30 s, then 10 min on ice in an ultrasonic disruptor. Samples were then incubated at −40°C for 1 h, followed by centrifugation at 12,000 rpm for 15 min at 4°C. The resulting supernatant was then utilized for LC-MS/MS measurements (Doppler et al., 2016).

### LC-MS/MS Analysis

LC-MS/MS analyses were performed using a UHPLC system (1290, Agilent Technologies) with a UPLC HSS T3 column (2.1 mm × 100 mm, 1.8 μm) coupled to a Q Exactive mass spectrometer (Orbitrap MS, Thermo). The mobile phase A consisted of 0.1% formic acid in water at the positive mode and 5 mmol/L ammonium acetate in water for the negative mode, and the mobile phase B was acetonitrile. The elution gradient was set as follows: 0–1 min, 1% B; 1–8 min, 1%–99% B; 8–10 min, 99% B; 10–10.1 min, 99%–1% B; 10.1–12 min, 1% B. The flow rate was set to 0.5 mL/min, while the injection volume was set to 2 μL. A QE mass spectrometer was utilized to acquire MS/MS spectra, with the collective mode set to information-dependent acquisition (IDA) in the acquisition software (Xcalibur 4.0.27, Thermo). In this mode, the acquisition software continuously evaluates the full scan MS spectrum. The ESI source conditions were set as follows: sheath gas flow rate as 45 Arb, aux gas flow rate as 15 Arb, capillary temperature 400°C, full MS resolution as 70000, MS/MS resolution as 17500, collision energy as 20/40/60 eV in NCE mode, spray Voltage as 4.0 kV (positive) or −3.6 kV (negative), respectively (Periannan, 2003).

### Statistical Analyses

The cell number, chlorophyll content, dry weight, and paramylon content of *E. gracilis* were analyzed using a parametric two-way analysis of variance (ANOVA) with treatment (co-cultivated and axenic) as the source of variations. All data fulfilled the assumptions of the parametric test and no data transformation was needed. The statistical analysis was carried out by SPSS 17.0 for Windows.

For the metabolomic study, after the raw data profiles were preprocessed, Student’s *t*-tests were utilized for univariate analysis, while principal component analysis (PCA) and orthogonal projections to latent structures discriminant analysis (OPLS-DA) were used for multivariate analysis. Variable importance in projection (VIP) score was combined with P-value to screen significant differential metabolites. In-house database and online databases (HMDB and Metlin) were applied in metabolite identification. Metabolites which were found to be statistically significantly different in different samples were then qualitatively analyzed based on relevant KEGG pathway and literature information (Mangalam et al., 2013).

## Results

### Effects of Co-cultivation on the Growth (Cell Number, Chlorophyll Content, Dry Weight) and Paramylon Content of *E. gracilis*

**FIGURE 1:**
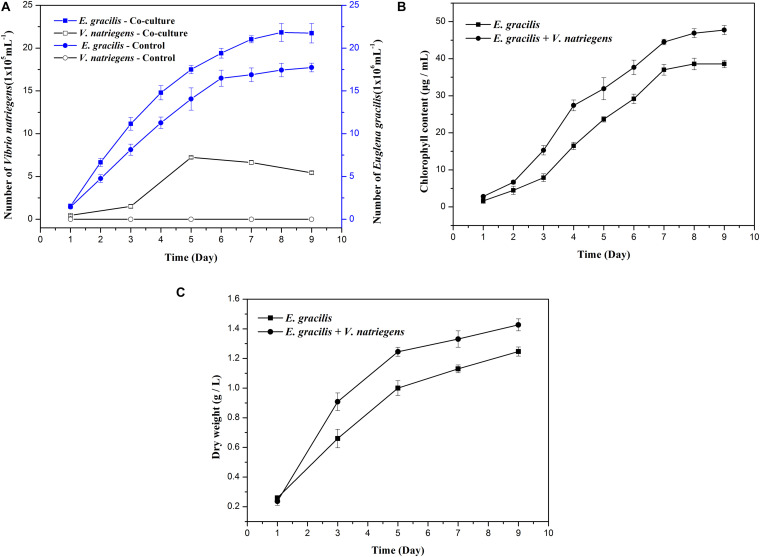
Effects of the co-cultivation system on the growth of *E. gracilis*. **(A)** Cell number of *E. gracilis* and *V. natriegens*. **(B)** Chlorophyll content. **(C)** Dry weight. There was an extremely significant effect of the treatment (co-cultivated and axenic) according to ANOVA test at the *p* < 0.0001 level for the three conditions (*F*-values are 229.557, 381.066, and 120.642, respectively).

When compared to the *E. gracilis* control, the co-cultivation group had significantly higher biomass and produced more paramylon. As shown in Figure 1A, the average growth rate of *E. gracilis* was faster when co-cultivated with *V. natriegens*, and the co-cultivation group not only entered the exponential phase approximately 24 h earlier than the control but also entered the stationary phase 24 h later. The average division speed of *E. gracilis* under the co-cultivation system was also much faster than that of the control. When the control entered the stationary phase after 6 days and the cell division slowed, the co-cultivation group continued to divide rapidly. After 9 days of cultivation, the cell number of the co-cultivation group was 23% higher than that of the control group. Interestingly, as shown in Figure 1B, the chlorophyll content of the co-cultivation group (47.73 μg/mL) in the late culture period (D9) increased by 23.75% compared with that of the control (38.57 μg/mL).

Changes in the number of *V. natriegens* cells in the co-cultivation system are shown in Figure 1A. *V. natriegens* grew slowly during D1–D3 of co-cultivation, and the number of bacteria began to increase sharply from D3 onward. On D5, the growth of *V. natriegens* entered into a stationary phase (7.23 x 10^5^ cells/mL), while the number of *V. natriegens* was 15.38 times and 1.33 times higher than that on D1 (0.47 x 10^5^ cells/mL) and D9 (5.43 x 10^5^ cells/mL), respectively. The *V. natriegens* cell count then began to decline, indicating that this species can exist symbiotically with *E. gracilis*, and its presence is correlated with higher *E. gracilis* growth rates.

**FIGURE 2:**
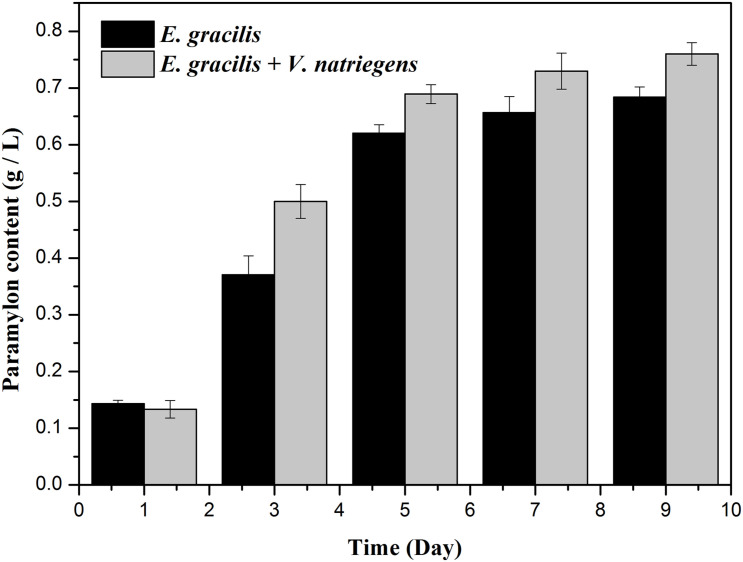
Effects of the co-cultivation system on active substance (paramylon content) accumulation of *E. gracilis*. There was an extremely significant effect of the treatment (co-cultivated and axenic) according to ANOVA test at the *p* < 0.0001 level for dry weight (*F*-value = 63.938).

As shown in Figures 1C, 2, both the dry weight and paramylon content increased in the two groups over time, but the dry weight and paramylon content in the co-cultivation group were significantly higher than that of the control group. On D9, dry weight and paramylon content of the co-cultivation group reached their maximum values, 1.42 and 0.76 g/L, respectively, which were approximately 15 and 12% higher than the control (1.24 and 0.68 g/L).

### Comparison of the Metabolic Changes in the Co-cultivation Group and Axenic *E. gracilis* Group

#### General Analysis of Metabolites

A total of 5165 metabolites were detected in the samples (Table 1), and 100 metabolites were identified and shown in Supplementary Data. Metabolites with *p*-values < 0.05 and VIP > 1 were considered differential metabolites between the two groups, and 339 metabolites were identified as passing these criteria. Among these significantly differentially expressed metabolites, 172 were upregulated and 167 were downregulated when comparing the co-cultivation group to the control group.

**TABLE 1:** The general and differential ion numbers in metabolomic results.

Mode	Total ion number	VIP > 1 ion number	*p*-value < 0.05 ion number	Up	Down
Pos	5165	1838	339	172	167
Neg	5259	1968	317	213	104

#### Multivariate Data Analysis

**FIGURE 3:**
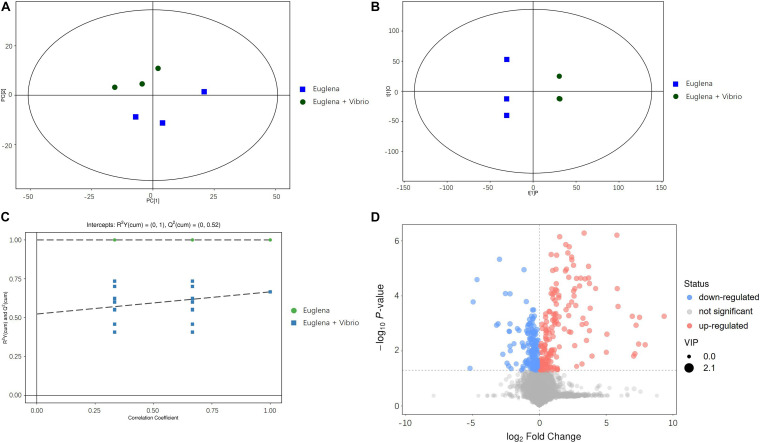
**(A)** PCA analysis of metabolomic profiles. **(B)** OPLS-DA scattered plots of metabolomic profiles. **(C)** OPLS-DA permutation plots of metabolomic profiles. **(D)** Volcano plots of metabolomic profiles.

Principal component analysis plots of the metabolomic profiles after 6 days of cultivation between the co-cultivation group and the control were generated, as shown in Figure 3A. All the samples were within the 95% confidence ellipses. Figure 3A indicates that a suitable distance was found between dispersion and aggregation samples when comparing the control and co-cultivation groups, suggesting that the two groups of samples have significant differences in the chemical composition of metabolites. OPLS-DA scatter plots are shown in Figure 3B. The two groups of samples were distinguished significantly and were both within the 95% confidence ellipses. The control and the co-cultivation group were distributed along the first principal component t [1] axis, with no crossover or overlap, indicating that the composition of the metabolites in the two groups was significantly different, and the extracellular metabolites of *E. gracilis* were affected by *V. natriegens.* The OPLS-DA permutation plots are shown in Figure 3C. R^2^Y was used to estimate the matching degree between the structured model and the Y data, and Q^2^ was utilized to judge the predictive ability of the structured model. The values of R^2^Y and Q^2^ were both higher than 0.05, indicating that the original model had a high degree of fit and high explanatory and predictive capabilities. After visualizing the differential metabolites in the form of volcano plots in Figure 3D (VIP > 1 and *p*-value < 0.05), it can be found that the differences between the control group and the co-cultivation group were significant.

### Significant Differential Metabolites

Metabolites with *p*-value < 0.05 were considered significant differential metabolites, and were the driving force of the separation shown in the models constructed previously (Table 2). When compared to the control, the co-cultivation group had significantly higher levels of methyl carbamate, ectoine, choline, methyl N-methylanthranilate, gentiatibetine, 4R-aminopentanoic acid and glu-val, while the levels of 3-butylpyridine, proline, sn-glycero-3-phosphocholine, N-butyl-1H-pyrazolo[3,4-d]pyrimidin-4-amine, and myosmine were lower.

**TABLE 2:** The differential metabolites and their indexes.

Metabolite	*p*-value	Fold change
Gentiatibetine	0.0005	1.39
4R-aminopentanoic acid	0.0006	124.23
sn-Glycero-3-phosphocholine	0.0010	0.12
Glu-Val	0.0012	151.38
Methyl N-methylanthranilate	0.0062	1.32
Proline	0.0065	0.55
Choline	0.0079	1.33
3-Butylpyridine	0.0101	0.67
Ectoine	0.0202	1.49
Methyl carbamate	0.0270	1.11
N-Butyl-1H-pyrazolo[3,4-d]pyrimidin-4-amine	0.0458	0.71
Myosmine	0.0493	0.40

### Correlation Analysis of Significant Differential Metabolites

**FIGURE 4:**
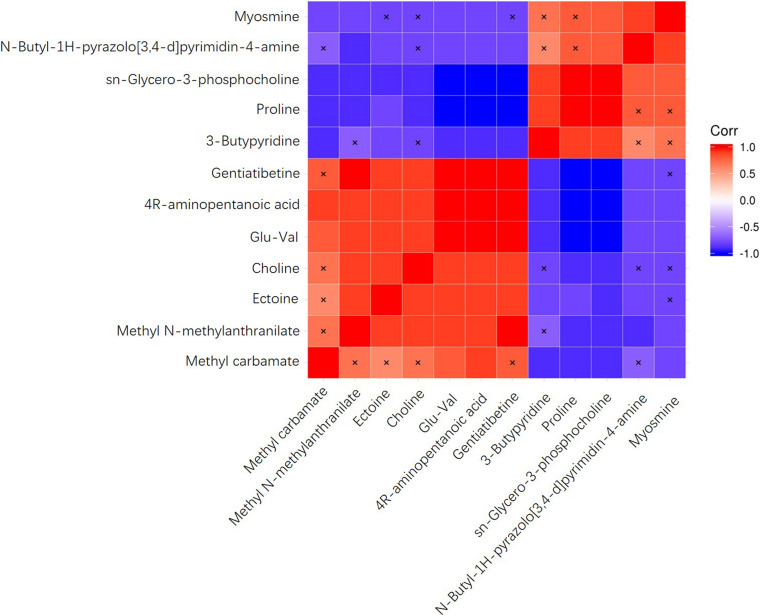
Heatmaps of correlation analysis plots of differential metabolites.

To investigate the correlation of significantly differential metabolites, heat maps were generated (Figure 4) using the Pearson’s correlation coefficient. Positive R values denote positive correlation while negative R values denote negative correlation. The metabolite 4R-aminopentanoic acid, for example, was positively correlated with methyl carbamate, methyl N-methylanthranilate, ectoine, glu-val, and gentiatibetine, with glu-val and gentiatibetine showing the highest R values. At the same time, this metabolite was negatively correlated with 3-butylpyridine, proline, sn-glycero-3-phosphocholine, N-butyl-1H-pyrazolo[3,4-d]pyrimidin-4-amine and myosmine, with proline and sn-glycero-3-phosphocholine showing the lowest R values.

### GO KEGG Analyses

In addition to multivariable analysis, metabolites were mapped to KEGG metabolic pathways for enrichment analysis (Table 3). Pathway enrichment analysis showed that “Glycine, serine and threonine metabolism,” “Glycerophospholipid metabolism,” and “ABC transporters” were statistically significantly enriched.

**TABLE 3:** The variation trends of differential metabolites in each enriched pathway.

Metabolites	Metabolic pathways		
	Glycine, serine and threonine metabolism	Glycerophospholipid metabolism	ABC transporters
Choline	↑	↑	↑
Ectoine	↑	–	↑
sn-Glycero-3-phosphocholine	–	↓	–
Proline	–	–	↓

## Discussion

### Growth (Cell Number, Chlorophyll, Dry Weight) and Paramylon Content Under Co-cultivation

Under the optimal microalgae-bacteria ratio and co-cultivation conditions, the growth of *E. gracilis* can be improved significantly by *V. natriegens*. In the co-cultivation group, *E. gracilis* entered its exponential phase earlier and the exponential phase lasted longer compared to the control. The co-cultivation group also had values of cell number, chlorophyll content, dry weight and paramylon content which were 123, 124, 115, and 112% of the control, respectively.

Taken together, these results indicate that *V. natriegens* had a positive influence on *E. gracilis* under optimum growth conditions, resulting in higher reproductive efficiency, increased biomass and higher production of bioactive materials accumulation in *E. gracilis.* When the diatom *Thalassiosira pseudonana* was co-cultivated with the bacteria *Dinoroseobacter shibae* at a 1:1 ratio, the metabolism of *T. pseudonana* was altered, but its overall growth rate was unchanged (Paul et al., 2013). When cyanobacteria *Microcystis aeruginosa* PCC 7806 and microalga *Desmodesmus subspicatus* were co-cultivated in a designated dialysis tubing, the presence of *M. aeruginosa* did not influence the growth of the microalga at the early logarithmic growth phase, while the microalga started to out-compete the co-cultivated bacteria during the exponential phase of growth (Omidi et al., 2019). These findings indicate that species, cultivation conditions and co-cultivation ratio can all influence the results of co-cultivation. There’re other ways to optimize the biomass and bioactive materials accumulation in microalgae, different approaches were reported depend on the different purposes (Chew et al., 2017). For instance, optimizing the algal photobioreactor (Cheah et al., 2020) or modifying algal particles (Cheng et al., 2019) for wastewater treatment, many researches were also done to explore sustainable ways to utilize algae in bioenergy production (Chia et al., 2018).

Previous studies have also shown that when *E. gracilis* was heterotrophically co-cultivated with *V. natriegens*, significant increases in biomass and paramylon content were found (Kim et al., 2019). Although this previous study found positive results from co-cultivation, it did not explore the possible mechanisms or effects on the metabolites present in the system. In the current study, we not only analyzed the metabolites of the co-cultivation culture system, but also confirmed that bacteria can live in an algae-dominant environment, which may work to exclude the presence of other unwanted bacteria.

### *V. natriegens* in the Co-cultivation System

Under the optimal microalgae-bacteria ratio and cultivation conditions of this study, a balance of oxygen, carbon dioxide and nutrient substances was established between *V. natriegens* and *E. gracilis*. Meanwhile, the cell density of *V. natriegens* increased as *E. gracilis*’ cell density increased, indicating that the dead cells of *V. natriegens* (which could have been providing nutrients) were not the main reason why the production of *E. gracilis* was increased. It is plausible that *V. natriegens* produced metabolites that positively influenced the growth of *E. gracilis.* Moreover, *V. natriegens* entered both the stationary and decline phases earlier than *E. gracilis*, indicating that *V. natriegens* had a shorter life cycle than *E. gracilis*, Therefore, subsequent addition of more bacteria could be considered if this strategy was applied in the actual production process.

### Metabolomics of the Co-cultivation Group

#### General Metabolomic Analysis

Principal component analysis and OPLS-DA plots both revealed that the control and co-cultivation samples broke out into two distinct groups, indicating the two groups had significantly different metabolite profiles. Despite these significant differences, similarities were also found. The main metabolites identified from both groups were of similar types and quantities, including phosphoric acid, 2,5-xylidine, dimethylimidazole and 2-Aminopyridine. Although the existence of *V. natriegens* changed the composition of metabolites to some extent, the two groups were still cultured in the same medium and laboratory conditions, which led to the existence of similarities between metabolite profiles.

Many active substances were found at high levels in both the control and co-cultivation samples. 2-aminopyridine is an inhibitor of the beta-secretase enzyme and is useful in the treatment of conditions such as Alzheimer’s disease (Coburn et al., 2011). 1-butylamine and 2,5-xylidine are mainly utilized for organic synthesis and are important intermediates for the synthesis of some pesticides and medicines (Cao et al., 2011; Kricka and Vernon, 2011), while 2,5-xylidine can also be used for the synthesis of disazo acid dyes (Dombchik, 1977). Dimethicone is widely used in skincare and hair products (Pellicoro et al., 2013) and is also swallowed prior to upper endoscopy procedures due to its ability to reduce the foam and bubbles in both the stomach and the duodenum to increase visibility. The discovery of these active substances indicates a significant potential for *E. gracilis* production (Bertoni et al., 1992).

#### Metabolites Which Are Related to the Proliferation of *E. gracilis*

The metabolite with the highest fold change (151) was glu-val. Glu-val belongs to the class of organic compounds known as peptides (Maehashi et al., 1999), and it is usually generated from proteolysis, which may be related to the downregulation of sn-glycero-3-phosphocholine and upregulation of choline. Choline is an important substrate involved in the synthesis of phosphatidylcholine (Li and Vance, 2008), which is necessary for the biosynthesis of the eukaryotic cell membrane (Chen et al., 2014). Since *V. natriegens* is a prokaryotic organism, the upregulation of phosphatidylcholine directly indicates the increase of *E. gracilis* membrane and further indicates an increase of *E. gracilis* biomass, which is in keeping with our analysis of biomass during co-cultivation. A higher quantity of *E. gracilis* cells would result in a higher rate of proteolysis, which may explain the increased level of glu-val in the co-cultivation group, as well as the downregulation of proline content. Interestingly, the mean value of glu-val in the co-cultivation group (0.0593) was much higher than that in the control (0.0004), even though both values were relatively small. This may be due to the generation of excess glu-val directly by *V. natriegens* or indirectly by *E. gracilis* under the influence of *V. natriegens*.

In the co-cultivation group, *V. natriegens* likely produced a significant amount of ectoine to adapt to the osmotic stress change in the medium (Teixidó et al., 2005). Ectoine is a solute that is accumulated by the halophilic or halotolerant microorganisms to prevent osmotic stress, which can also protect non-halophilic cells (Fallet et al., 2010). Meanwhile, studies have shown that ectoine causes an increase in the expression level of the ABC transporter substrate-binding protein EhuB (Richter et al., 2019). It therefore seems likely that uptake of ectoine by *E. gracilis* caused the upregulation of metabolites which are imported by ABC transporters.

Ectoine is widely used in the plant industry, due to its ability to accelerate the enzymatic conversion of triglycerides in biodiesel synthesis (Wang and Zhang, 2010). Ectoine can also increase cellular tolerance to high salt concentration, which can block chlorophyll synthesis in plants and microalgae (Pinheiro et al., 2008). The mean value of ectoine in the co-cultivation group (0.0104) was slightly higher than that in the control group (0.0070), likely because ectoine was either generated by *V. natriegens* or *E. gracilis* under the influence of *V. natriegens.*

#### Metabolites Related to the Value of Large-Scale Exploitation of *E. gracilis*

4R-aminopentanoic acid content in the co-cultivation group was higher than that in the control group (124-fold). This study represents the first time that 4R-aminopentanoic acid has been found in microalgae. In pharmacology, derivatives of this compound can be used as GABA transaminase and NEP inhibitors, which act as anticonvulsants (Callery et al., 1982). The synthesis of 4-aminopentanoic acid mainly depends on artificial means (Silverman and Levy, 2002), but with the discovery of the ability of *E. gracilis* to produce 4-aminopentanoic acid opens up new possibilities for producing this important compound. Similar to glu-val, the mean value of 4R-aminopentanoic acid in the co-cultivation group (6.6746) was much higher than that in the control group (0.0537), likely because this metabolite was produced by *V. natriegens* or *E. gracilis* under the influence of *V. natriegens.*

Some of the metabolites identified in this study are of commercial finterest, potentially increasing the value of *E. gracilis* cultivation. For example, methyl N-methylanthranilate and methyl carbamate were both upregulated in the co-cultivation group. Methyl N-methylanthranilate is a natural fragrance that can be found in flowers and fruit (Mookherjee et al., 1990), which is also added to wine as an aroma constituent (Nelson et al., 1977). Additionally, it appears in several essential oils, such as neroli and bergamot (Taupp et al., 2005). Myosmine is a minor tobacco alkaloid which is downregulated during co-cultivation (Zwickenpflug and Tyroller, 2006). This compound has been shown to be a potential risk factor for the development of esophageal adenocarcinoma (Vogt et al., 2006), and its downregulation could improve the safety of *E. gracilis* consumption.

In a previous study, it was proposed that *V. natriegens* increased the production of *E. gracilis* through the impact of indole-3-acetic acid (IAA) (Kim et al., 2019), but we were unable to confirm this result. This previous study also noted that the IAA produced by *V. natriegens* played a significant role in the positive growth regulation of *E. gracilis*, but the IAA concentration in our study was too low to be detected in the differential metabolite analysis.

#### Pathway Enrichment and KEGG Analyses

Choline and ectoine are known to participate in glycine, serine and threonine metabolism. Choline is a downstream product of serine, which is derived from 3P-D-glycerate, while 3P-D-glycerate is a derivative of glycolysis, and glycine is derived from serine. Threonine can only be synthesized by bacteria and plants, but not by animals (Shaul and Galili, 1993). Threonine is derived from aspartic acid (Szczesiul and Wampler, 2002).

Two metabolites of sn-glycero-3-phosphocholine and choline participate in glycerophospholipid metabolism (Tocher, 1995). These two metabolites are the downstream products of phosphatidylcholine (Li and Vance, 2008), and choline is a precursor for the synthesis of phosphatidylcholine (Vance and Adeli, 2008). Since phosphatidylcholine generally does not exist in prokaryotes (Fagone and Jackowski, 2013), it can be inferred that the change in this pathway is originated in *E. gracilis.* It is worth noting that, similar to the differential metabolite ectoine, the metabolomic results show that the mean values of choline in the co-cultivation group (0.4197) and in the control group (0.0316) are both low. Therefore, it seems likely that this metabolite was produced by either microalgal or bacterial cells under the influence of *V. natriegens*, and may have played a role in the increased growth rate of *E. gracilis*.

ABC transporter activity influences the levels of proline, osmo-protectants and histidine compounds (Boncompagni et al., 2000). These transporters are membrane integral proteins that use the energy generated by hydrolyzing ATP to actively transport carbohydrates, amino acids, peptides, proteins and various cellular metabolites (Rees et al., 2009). They can be found in the cell membranes of both eukaryotes and prokaryotes (Pohl et al., 2005), and the changes to their expression significantly impact the transport capacity of microbial membranes in the co-cultivation system.

## Conclusion

Effects of *E. gracilis* and *V. natriegens* co-cultivation on microalgal physiological characteristics, cellular metabolites and metabolic networks revealed that the biomass and paramylon content of *E. gracilis* was enhanced by *V. natriegens*. A total of 339 differential metabolites were found, including economically important metabolites such as choline, ectoine, 4R-aminopentanoic acid, methyl N-methylanthranilate and methyl carbamate. This study represents the first comprehensive metabolomic study of culture medium involving a microalgae-bacteria co-cultivation system. Overall, this study significantly increases the understanding of microalgae-bacteria co-cultivation systems and provides a number of new avenues to explore for improving the mass production of *E. gracilis*.

## Data Availability Statement

The original contributions presented in the study are included in the article/Supplementary Material, further inquiries can be directed to the corresponding author/s.

## Author Contributions

JH and JW contributed to the conceptualization, methodology, writing (review and editing), formal analysis, and investigation. YO and SC contributed to the investigation, data curation, writing (original draft), and formal analysis. LZ contributed to the writing (review and editing). YS contributed to the investigation. AL contributed to the data curation and writing (review and editing). All authors contributed to the article and approved the submitted version.

## Conflict of Interest

The authors declare that the research was conducted in the absence of any commercial or financial relationships that could be construed as a potential conflict of interest.

## Footnotes

1 https://www.ccap.ac.uk/

## Abbreviations

IAA: indole-3-acetic acid
KEGG: Kyoto Encyclopedia of Genes and Genomes
OPLS-DA: orthogonal projections to latent structures discriminant analysis
PCA: principal component analysis
VIP: variable importance in projection.
