# Supplementary material for: Global Metabolomics Reveals That Vibrio natriegens Enhances the Growth and Paramylon Synthesis of Euglena gracilis
Source: Front Bioeng Biotechnol. 2021 Mar 31;9:652021. doi: 10.3389/fbioe.2021.652021 (PMC8044410; doi:10.3389/fbioe.2021.652021)
Supplement: Supplementary file 1 [file Table_1.DOCX]

Supplementary Material

**Supplementary Table.** Identified metabolites

| No. | Metabolites | VIP | P-value | Mean EG | Mean CG |
| --- | --- | --- | --- | --- | --- |
| 1 | 4R-aminopentanoic acid | 2.13 | 0.0006 | 6.68 | 0.05 |
| 2 | Phosphoric acid | 0.81 | 0.4694 | 2.20 | 2.04 |
| 3 | 2,5-Xylidine | 1.12 | 0.2732 | 2.86 | 2.97 |
| 4 | Proline | 2.12 | 0.0065 | 0.18 | 0.33 |
| 5 | 2,2',2''-Nitrilotriethanol | 0.34 | 0.8505 | 0.21 | 0.23 |
| 6 | Cytosine | 0.31 | 0.7930 | 0.72 | 0.63 |
| 7 | Choline | 1.94 | 0.0079 | 0.42 | 0.32 |
| 8 | Met His Lys | 0.57 | 0.5643 | 0.08 | 0.07 |
| 9 | 4-Isopropylaniline | 0.57 | 0.6379 | 0.35 | 0.43 |
| 10 | Sphinganine | 0.31 | 0.8157 | 0.14 | 0.14 |
| 11 | 1-Butylamine | 0.85 | 0.4921 | 2.04 | 1.68 |
| 12 | 2-Pyrrolidinone | 0.33 | 0.6704 | 0.06 | 0.07 |
| 13 | 3-METHYLHISTAMINE | 0.19 | 0.8825 | 1.36 | 1.48 |
| 14 | Carnitine | 1.65 | 0.0739 | 0.08 | 0.10 |
| 15 | 1-Pyrroline | 1.4 | 0.1186 | 0.07 | 0.17 |
| 16 | Calcium L-aspartate | 0.98 | 0.3631 | 1.82 | 0.76 |
| 17 | 2'-O-Methyladenosine | 0.54 | 0.6210 | 0.02 | 0.02 |
| 18 | Oleoyl Ethanolamide-d2 | 0.65 | 0.5839 | 0.19 | 0.20 |
| 19 | Histamine | 0.55 | 0.6922 | 2.15 | 1.57 |
| 20 | Acetone cyanohydrin | 1.03 | 0.3492 | 0.03 | 0.04 |
| 21 | 2-Aminopyridine | 1.22 | 0.2376 | 2.43 | 1.79 |
| 22 | 3-Butylpyridine | 1.97 | 0.0101 | 0.17 | 0.25 |
| 23 | N-Butyl-1H-pyrazolo[3,4-d]pyrimidin-4-amine | 1.74 | 0.0458 | 0.34 | 0.48 |
| 24 | N,N-Dimethylaniline | 1.56 | 0.0984 | 0.43 | 0.32 |
| 25 | 3-Hydroxypyridine | 0.67 | 0.6312 | 0.04 | 0.03 |
| 26 | Isopropylpyrazine | 0.94 | 0.3825 | 0.22 | 0.20 |
| 27 | D-2-Aminobutyric acid | 1.63 | 0.0697 | 0.13 | 0.17 |
| 28 | DEA | 0.88 | 0.4740 | 0.10 | 0.08 |
| 29 | 2-Imino-4-methylpiperidine | 0.38 | 0.7019 | 0.10 | 0.11 |
| 30 | Didesmethylpheniramine | 0.11 | 0.9093 | 0.03 | 0.03 |
| 31 | Nicotinic acid | 0.46 | 0.7032 | 0.14 | 0.14 |
| 32 | beta-Aminopropionitrile | 0.78 | 0.5067 | 0.15 | 0.16 |
| 33 | Gyromitrin | 0.85 | 0.5749 | 0.80 | 0.62 |
| 34 | Pyrrolidine | 0.97 | 0.3639 | 0.01 | 0.01 |
| 35 | 2,5-Dihydro-2,4,5-trimethyloxazole | 1.08 | 0.2996 | 0.21 | 0.17 |
| 36 | 1-Methylpiperazine | 1.38 | 0.1123 | 0.22 | 0.11 |
| 37 | Pantothenic acid | 1.73 | 0.0506 | 0.02 | 0.02 |
| 38 | sn-Glycero-3-phosphocholine | 2.14 | 0.0010 | 0.01 | 0.05 |
| 39 | Ectoine | 1.9 | 0.0202 | 0.01 | 0.01 |
| 40 | Tacrine | 0.59 | 0.6315 | 0.05 | 0.04 |
| 41 | 2,6-Dimethylpyrazine | 0.36 | 0.8503 | 0.06 | 0.06 |
| 42 | N,N-dimethyl-Safingol | 0.06 | 0.9490 | 1.43 | 1.43 |
| 43 | DDAO | 0.78 | 0.5378 | 0.04 | 0.04 |
| 44 | 7-alpha-D-Ribosyladenine 5'-phosphate | 0.71 | 0.5056 | 0.01 | 0.01 |
| 45 | Capsi-amide | 0.62 | 0.6069 | 0.03 | 0.03 |
| 46 | Spisulosine | 1.11 | 0.2569 | 0.03 | 0.05 |
| 47 | Erucamide | 1.2 | 0.2052 | 0.09 | 0.17 |
| 48 | Anandamide (20:l, n-9) | 0.63 | 0.5712 | 0.15 | 0.15 |
| 49 | Thalassemine | 1.5 | 0.1303 | 0.03 | 0.02 |
| 50 | Acetylcarnitine | 0.98 | 0.3574 | 0.04 | 0.04 |
| 51 | Benzenesulfonamide | 0.32 | 0.9728 | 0.70 | 0.71 |
| 52 | Oleamide | 0.29 | 0.6563 | 0.05 | 0.04 |
| 53 | 4-Amino-5-hydroxymethyl-2-methylpyrimidine | 1.66 | 0.0897 | 0.34 | 0.21 |
| 54 | Dimethicone | 1.26 | 0.1729 | 1.20 | 0.73 |
| 55 | L-Histidinal | 0.36 | 0.7781 | 1.09 | 0.93 |
| 56 | 3-Methylamino-L-alanine | 1.21 | 0.2776 | 0.08 | 0.10 |
| 57 | Nicotinamide | 0.82 | 0.4901 | 0.04 | 0.04 |
| 58 | 1-methylpyrrole | 1.05 | 0.3235 | 0.09 | 0.05 |
| 59 | Picolinamide | 1.14 | 0.3253 | 0.14 | 0.13 |
| 60 | Methyl N-methylanthranilate | 1.98 | 0.0062 | 0.14 | 0.11 |
| 61 | N-Nitrosodiethylamine | 0.72 | 0.4053 | 0.07 | 0.08 |
| 62 | (2-Methoxyethoxy)propanoic acid | 0.01 | 0.7956 | 1.23 | 1.53 |
| 63 | Gentiatibetine | 2.1 | 0.0005 | 0.48 | 0.35 |
| 64 | Monodesmethylpheniramine | 0.71 | 0.5212 | 0.06 | 0.06 |
| 65 | N,N,N-trimethyl-sphingosine | 0.88 | 0.4142 | 0.03 | 0.03 |
| 66 | Tri(butoxyethyl)phosphate | 0.29 | 0.7756 | 0.12 | 0.12 |
| 67 | Dimethylimidazole | 1.64 | 0.0749 | 3.71 | 2.65 |
| 68 | Lys Lys Thr | 0.08 | 0.9112 | 0.02 | 0.02 |
| 69 | Digitalose | 0.46 | 0.6695 | 0.60 | 0.64 |
| 70 | Pro Val Gly | 0.31 | 0.8209 | 0.05 | 0.04 |
| 71 | Ile Ala Phe | 0.99 | 0.3639 | 0.03 | 0.03 |
| 72 | Trimethylpyrazine | 0.25 | 0.8574 | 0.41 | 0.37 |
| 73 | N-Palmitoyl Glycine | 0.39 | 0.7481 | 0.02 | 0.02 |
| 74 | 1-Deoxy-D-glucitol | 0.02 | 0.6656 | 0.31 | 0.25 |
| 75 | Phytosphingosine | 0.51 | 0.5987 | 0.01 | 0.01 |
| 76 | N-Nitrosomethylvinylamine | 1.15 | 0.2023 | 0.18 | 0.11 |
| 77 | N,N-Dimethyldodecylamine N-oxide | 0.79 | 0.5342 | 0.26 | 0.23 |
| 78 | Tetraacetylethylenediamine | 0.62 | 0.4857 | 0.18 | 0.11 |
| 79 | Methyl carbamate | 1.85 | 0.0270 | 0.22 | 0.19 |
| 80 | His | 0.72 | 0.5165 | 0.24 | 0.25 |
| 81 | NERYL ACETATE | 1.01 | 0.3687 | 0.19 | 0.07 |
| 82 | ALVERINE | 0.99 | 0.3468 | 0.02 | 0.02 |
| 83 | Sakacin P | 1.04 | 0.3076 | 0.12 | 0.15 |
| 84 | Val Gly Pro | 0.77 | 0.5380 | 0.10 | 0.08 |
| 85 | Myosmine | 1.58 | 0.0493 | 0.07 | 0.18 |
| 86 | Glu-Val | 2.09 | 0.0012 | 0.06 | 0.00 |
| 87 | Trp-P-1 | 0.81 | 0.4094 | 0.05 | 0.03 |
| 88 | Arene oxide | 1.17 | 0.2687 | 0.07 | 0.06 |
| 89 | Myristoyl-EA | 1.1 | 0.3014 | 0.02 | 0.03 |
| 90 | DL-Histidinol | 1.25 | 0.1721 | 0.44 | 0.21 |
| 91 | Aniline | 0.67 | 0.5279 | 0.05 | 0.05 |
| 92 | 3,3-Dibromo-2-n-hexylacrylic acid | 0.44 | 0.6920 | 0.46 | 0.46 |
| 93 | Gly Val Pro | 0.77 | 0.4249 | 0.08 | 0.14 |
| 94 | Phenol | 0.54 | 0.5534 | 0.03 | 0.02 |
| 95 | 2-Aminobut-2-enoate | 0.55 | 0.7381 | 0.04 | 0.04 |
| 96 | Cucurbitine | 0.52 | 0.5667 | 0.08 | 0.09 |
| 97 | Octylamine | 1.57 | 0.0962 | 0.28 | 0.27 |
| 98 | Triethylcitrate | 1.48 | 0.1352 | 0.03 | 0.02 |
| 99 | C16 Sphinganine | 0.54 | 0.6694 | 0.85 | 0.92 |
| 100 | Eicosanoyl-EA | 0.25 | 0.8449 | 0.45 | 0.45 |

Note: MEAN XX means the relative quantitative value of an experimental group of the substance in the comparison.
